# (η^5^-Cyclo­penta­dien­yl)bis­(triphenyl­phosphane)cobalt(I)–toluene–*n*-hexane (1/0.20/0.25)

**DOI:** 10.1107/S1600536808042268

**Published:** 2008-12-17

**Authors:** Marko Hapke, Anke Spannenberg

**Affiliations:** a Leibniz-Institut für Katalyse e. V. an der Universität Rostock, Albert-Einstein-Strasse 29a, 18059 Rostock, Germany

## Abstract

The title compound, [Co(C_5_H_5_)(C_18_H_15_P)_2_]·0.2C_7_H_8_·0.25C_6_H_14_, was synthesized by the reaction of cobaltocene, Cp_2_Co, with elemental lithium in tetra­hydro­furan in the presence of two equivalents of PPh_3_. The mol­ecular structure displays a cobalt(I) center in a distorted trigonal-planar coordination environment, with one Cp and two phosphane ligands. There are two crystallographically independent mol­ecules in the asymmetric unit besides the disordered solvent molecules.

## Related literature

For a general background and synthetic procedure, see: Jonas *et al.* (1981 ▶, 1983 ▶). For derivatives with chiral Cp-ligands, see: Gutnov *et al.* (2003 ▶, 2004 ▶). For the structurally related compound CpCo(PEt_3_)_2_, see: Harlow *et al.* (1983 ▶).
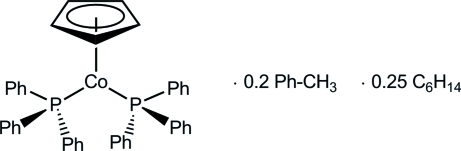


## Experimental

### 

#### Crystal data

[Co(C_5_H_5_)(C_18_H_15_P)_2_]·0.2C_7_H_8_·0.25C_6_H_14_
*M* *_r_* = 688.53
Triclinic, 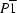

*a* = 10.5496 (3) Å
*b* = 18.2220 (5) Å
*c* = 19.0367 (5) Å
α = 86.543 (2)°
β = 75.093 (2)°
γ = 89.691 (2)°
*V* = 3529.7 (2) Å^3^
*Z* = 4
Mo *K*α radiation
μ = 0.61 mm^−1^
*T* = 200 (2) K
0.5 × 0.3 × 0.3 mm

#### Data collection

Stoe IPDSII diffractometer
Absorption correction: numerical (*X-SHAPE*; Stoe & Cie, 2005 ▶) *T*_min_ = 0.787, *T*_max_ = 0.868
48156 measured reflections
13135 independent reflections
9423 reflections with *I* > 2σ(*I*)
*R*_int_ = 0.032

#### Refinement

*R*[*F* ^2^ > 2σ(*F* ^2^)] = 0.032
*wR*(*F* ^2^) = 0.080
*S* = 0.92
13135 reflections
829 parameters
6 restraints
H-atom parameters constrained
Δρ_max_ = 0.71 e Å^−3^
Δρ_min_ = −0.26 e Å^−3^

### 

Data collection: *X-AREA* (Stoe & Cie, 2005 ▶); cell refinement: *X-AREA*; data reduction: *X-RED* (Stoe & Cie, 2005 ▶); program(s) used to solve structure: *SHELXS97* (Sheldrick, 2008 ▶); program(s) used to refine structure: *SHELXL97* (Sheldrick, 2008 ▶); molecular graphics: *SHELXTL* (Sheldrick, 2008 ▶); software used to prepare material for publication: *SHELXTL*.

## Supplementary Material

Crystal structure: contains datablocks I, global. DOI: 10.1107/S1600536808042268/im2092sup1.cif

Structure factors: contains datablocks I. DOI: 10.1107/S1600536808042268/im2092Isup2.hkl

Additional supplementary materials:  crystallographic information; 3D view; checkCIF report

## Figures and Tables

**Fig. 1.**
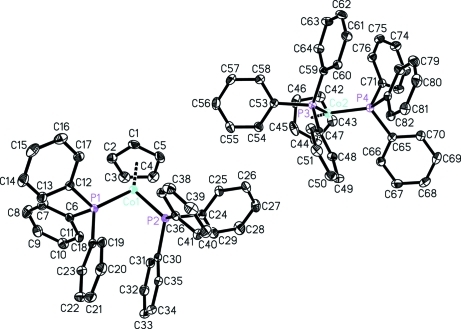
The molecular structure of the title compound showing the atom-labelling scheme (except C37, C52, C72, C73, C77 and C78). Thermal ellipsoids are drawn at the 30% probability level. Hydrogen atoms and solvent molecules are omitted for clarity.

## supplementary crystallographic information

### Comment

The "CpCo"-fragment is a frequently used structural motif in catalytic reactions involving organocobalt species. Usually the "CpCo" fragment is stabilized by two donor ligands and a number of stable and commercially available complexes are known. Well known examples are the so-called Jonas complex CpCo(ethylene)_2_ (Jonas *et al.*, 1981) and the carbonyl complex Cp(Co)(CO)_2_, which has frequently been used as precursor in cobalt-catalysed cycloaddition reactions. We have prepared CpCo(PPh_3_)_2_ as a precursor compound for the synthesis of different cobalt complexes and the screening of catalytic reactions. Recently, we adopted a novel synthethic procedure for CpCo(PPh_3_)_2_ by reacting cobaltocene, Cp_2_Co, with elemental lithium in THF in the presence of 2 equivalents of PPh_3_, following a general procedure that has been published (Jonas *et al.*, 1983). The synthesis of chiral cyclopentadienyl (Cp*) cobalt complexes, which were used as catalysts in atroposelective [2 + 2+2] cycloaddition reactions, frequently includes triphenylphosphane-stabilized Cp*Co-intermediates (Gutnov *et al.*, 2003, 2004). In the synthetic sequence CoCl(PPh_3_)_3_ is reacted with Cp*Li, yielding the Cp*Co(PPh_3_)_2_ compound, that can readily be converted to the cyclooctadiene complex.

Here we prepared the unsubstituted bisphosphane complex by a reductive methodology, using lithium as the reducing agent for cobaltocene in the presence of the triphenylphosphane under very mild and convenient reaction conditions.

The structure of the title complex contains two molecules in the asymmetric unit besides *n*-hexane and toluene as lattice solvents with occupancies 0.5:0.4. The P—Co—P angle is 99.57 (3) and 99.67 (2) %, respectively, with one phenyl group of each phosphane being nearly parallel to each other (Fig.1).

### Experimental

Cobaltocene (2 g, 10.58 mmol), triphenylphosphane (5.55 g, 21.15 mmol) and freshly cut lithium (74 mg, 10.69 mmol) were weighted into a Schlenk flask in the glove-box. The reaction flask was connected to a Schlenk line outside the box and while cooling to -10 °C dry THF was added. After short time a colour change to deep red-violet was observed in the reaction flask and after 5 h stirring at a temperature between -10 to 0 °C the lithium has disappeared and the solution became markedly more viscous. THF was removed *in vacuo*. The residue was dissolved in toluene and filtrated over a G4 Schlenk filter frit. The filtrate was evaporated until only *ca* 25 ml of toluene were left. The same amount of *n*-hexane was added and the solution stored in the freezer. After crystallization of the product, the solid was filtered off with a Schlenk filter frit, washed with *n*-hexane and dried in high vacuo to yield 3.8 g of pure complex. The identity of the product was proven by ^1^H, ^13^C and ^31^P NMR.

### Refinement

All fully occupied non-hydrogen atoms are refined anisotropically. Atoms of solvent molecules are refined isotropically with occupancies 0.5:0.4 (*n*-hexane:toluene). All H atoms were placed in idealized positions with d(C—H) = 0.99 (CH_2_), 0.98 (CH_3_) and 0.95 Å (CH) and refined using a riding model with *U*_iso_(H) fixed at 1.5 *U*_eq_(C) for CH_3_ and 1.2 *U*_eq_(C) for CH_2_ and CH.

### Crystal data

**Table:**

[Co(C_5_H_5_)(C_18_H_15_P)_2_]·0.2C_7_H_8_·0.25C_6_H_14_	*Z* = 4
*M**_r_* = 688.53	*F*(000) = 1442
Triclinic, *P*1	*D*_x_ = 1.296 Mg m^−^^3^
*a* = 10.5496 (3) Å	Mo *K*α radiation, λ = 0.71073 Å
*b* = 18.2220 (5) Å	Cell parameters from 41669 reflections
*c* = 19.0367 (5) Å	θ = 1.5–27.2°
α = 86.543 (2)°	µ = 0.61 mm^−^^1^
β = 75.093 (2)°	*T* = 200 K
γ = 89.691 (2)°	Prism, dark-purple
*V* = 3529.7 (2) Å^3^	0.5 × 0.3 × 0.3 mm

### Data collection

**Table:**

Stoe IPDSII diffractometer	13135 independent reflections
Radiation source: fine-focus sealed tube	9423 reflections with *I* > 2σ(*I*)
graphite	*R*_int_ = 0.032
rotation method scans	θ_max_ = 25.5°, θ_min_ = 1.5°
Absorption correction: numerical (*X-SHAPE*; Stoe & Cie, 2005)	*h* = −12→12
*T*_min_ = 0.787, *T*_max_ = 0.868	*k* = −22→22
48156 measured reflections	*l* = −23→23

### Refinement

**Table:**

Refinement on *F*^2^	Primary atom site location: structure-invariant direct methods
Least-squares matrix: full	Secondary atom site location: difference Fourier map
*R*[*F*^2^ > 2σ(*F*^2^)] = 0.032	Hydrogen site location: inferred from neighbouring sites
*wR*(*F*^2^) = 0.080	H-atom parameters constrained
*S* = 0.92	*w* = 1/[σ^2^(*F*_o_^2^) + (0.0468*P*)^2^] where *P* = (*F*_o_^2^ + 2*F*_c_^2^)/3
13135 reflections	(Δ/σ)_max_ = 0.001
829 parameters	Δρ_max_ = 0.71 e Å^−^^3^
6 restraints	Δρ_min_ = −0.26 e Å^−^^3^

### Special details

**Table:**

Geometry. All e.s.d.'s (except the e.s.d. in the dihedral angle between two l.s. planes) are estimated using the full covariance matrix. The cell e.s.d.'s are taken into account individually in the estimation of e.s.d.'s in distances, angles and torsion angles; correlations between e.s.d.'s in cell parameters are only used when they are defined by crystal symmetry. An approximate (isotropic) treatment of cell e.s.d.'s is used for estimating e.s.d.'s involving l.s. planes.
Refinement. Refinement of *F*^2^ against ALL reflections. The weighted *R*-factor *wR* and goodness of fit *S* are based on *F*^2^, conventional *R*-factors *R* are based on *F*, with *F* set to zero for negative *F*^2^. The threshold expression of *F*^2^ > σ(*F*^2^) is used only for calculating *R*-factors(gt) *etc*. and is not relevant to the choice of reflections for refinement. *R*-factors based on *F*^2^ are statistically about twice as large as those based on *F*, and *R*- factors based on ALL data will be even larger.

### Fractional atomic coordinates and isotropic or equivalent isotropic displacement parameters (Å^2^)

**Table:**

	*x*	*y*	*z*	*U*_iso_*/*U*_eq_	Occ. (<1)
C83	0.6309 (8)	0.0938 (4)	0.1203 (4)	0.083 (2)*	0.50
H83A	0.5842	0.0773	0.0856	0.125*	0.50
H83B	0.7092	0.1221	0.0938	0.125*	0.50
H83C	0.5732	0.1250	0.1548	0.125*	0.50
C85	0.7389 (8)	0.0435 (4)	0.2121 (4)	0.072 (2)*	0.50
H85A	0.6931	0.0822	0.2432	0.087*	0.50
H85B	0.8270	0.0631	0.1863	0.087*	0.50
C86	0.7557 (9)	−0.0182 (4)	0.2587 (4)	0.085 (2)*	0.50
H86A	0.6669	−0.0394	0.2797	0.102*	0.50
H86B	0.8049	−0.0548	0.2261	0.102*	0.50
C87	0.8154 (12)	−0.0173 (5)	0.3178 (6)	0.111 (3)*	0.50
H87A	0.7874	0.0280	0.3436	0.133*	0.50
H87B	0.9116	−0.0135	0.2972	0.133*	0.50
C88	0.7890 (10)	−0.0784 (5)	0.3703 (5)	0.109 (3)*	0.50
H88A	0.8384	−0.0723	0.4067	0.163*	0.50
H88B	0.8156	−0.1239	0.3459	0.163*	0.50
H88C	0.6950	−0.0807	0.3943	0.163*	0.50
C84	0.6687 (6)	0.0315 (3)	0.1589 (3)	0.1200 (19)*	0.90
H84A	0.7232	0.0000	0.1225	0.144*	0.50
H84B	0.5881	0.0032	0.1834	0.144*	0.50
H84C	0.6265	−0.0101	0.1434	0.180*	0.40
H84D	0.7396	0.0513	0.1182	0.180*	0.40
H84E	0.6038	0.0699	0.1743	0.180*	0.40
C89	0.7259 (8)	0.0056 (5)	0.2234 (4)	0.088 (3)*	0.40
C90	0.7894 (9)	0.0593 (4)	0.2510 (5)	0.121 (4)*	0.40
H90	0.7929	0.1086	0.2314	0.145*	0.40
C91	0.8479 (9)	0.0407 (5)	0.3072 (5)	0.125 (4)*	0.40
H91	0.8913	0.0774	0.3260	0.150*	0.40
C92	0.8427 (9)	−0.0315 (5)	0.3358 (5)	0.112 (5)*	0.40
H92	0.8827	−0.0442	0.3742	0.134*	0.40
C93	0.7792 (10)	−0.0852 (4)	0.3082 (5)	0.143 (5)*	0.40
H93	0.7757	−0.1346	0.3278	0.172*	0.40
C94	0.7208 (8)	−0.0667 (4)	0.2520 (5)	0.106 (4)*	0.40
H94	0.6774	−0.1034	0.2332	0.128*	0.40
C1	0.1308 (3)	0.75684 (15)	0.32133 (13)	0.0414 (6)	
H1A	0.1820	0.7857	0.2808	0.050*	
C2	0.0437 (2)	0.78319 (14)	0.38396 (14)	0.0396 (6)	
H2A	0.0298	0.8335	0.3940	0.047*	
C3	−0.0194 (2)	0.72283 (15)	0.42916 (15)	0.0423 (6)	
H3A	−0.0855	0.7252	0.4735	0.051*	
C4	0.0333 (3)	0.65879 (15)	0.39653 (16)	0.0457 (7)	
H4A	0.0105	0.6098	0.4153	0.055*	
C5	0.1271 (3)	0.68004 (16)	0.33048 (15)	0.0456 (7)	
H5A	0.1788	0.6475	0.2978	0.055*	
C6	0.0772 (2)	0.83076 (12)	0.54939 (11)	0.0284 (5)	
C7	0.0236 (2)	0.90057 (14)	0.54925 (14)	0.0401 (6)	
H7A	0.0667	0.9378	0.5144	0.048*	
C8	−0.0928 (3)	0.91630 (15)	0.59972 (16)	0.0501 (7)	
H8A	−0.1291	0.9641	0.5987	0.060*	
C9	−0.1557 (3)	0.86332 (15)	0.65107 (14)	0.0436 (6)	
H9A	−0.2345	0.8746	0.6859	0.052*	
C10	−0.1042 (3)	0.79381 (14)	0.65189 (14)	0.0415 (6)	
H10B	−0.1471	0.7571	0.6875	0.050*	
C11	0.0098 (2)	0.77739 (13)	0.60099 (13)	0.0368 (6)	
H11A	0.0430	0.7288	0.6011	0.044*	
C12	0.2946 (2)	0.89196 (12)	0.43700 (12)	0.0287 (5)	
C13	0.3290 (2)	0.94745 (12)	0.47616 (14)	0.0368 (6)	
H13A	0.3110	0.9411	0.5277	0.044*	
C14	0.3890 (3)	1.01168 (14)	0.44071 (16)	0.0453 (6)	
H14A	0.4115	1.0493	0.4679	0.054*	
C15	0.4161 (3)	1.02094 (14)	0.36620 (16)	0.0473 (7)	
H15A	0.4581	1.0648	0.3419	0.057*	
C16	0.3827 (3)	0.96710 (15)	0.32700 (15)	0.0445 (6)	
H16A	0.4008	0.9741	0.2755	0.053*	
C17	0.3227 (2)	0.90247 (13)	0.36169 (13)	0.0348 (5)	
H17A	0.3007	0.8653	0.3339	0.042*	
C18	0.3443 (2)	0.79113 (11)	0.53970 (12)	0.0280 (5)	
C19	0.4773 (2)	0.79189 (12)	0.50431 (13)	0.0338 (5)	
H19A	0.5039	0.7978	0.4527	0.041*	
C20	0.5716 (3)	0.78416 (14)	0.54290 (16)	0.0439 (6)	
H20A	0.6620	0.7845	0.5178	0.053*	
C21	0.5341 (3)	0.77591 (14)	0.61804 (16)	0.0487 (7)	
H21A	0.5983	0.7698	0.6448	0.058*	
C22	0.4039 (3)	0.77658 (15)	0.65349 (15)	0.0479 (7)	
H22A	0.3781	0.7720	0.7052	0.057*	
C23	0.3090 (3)	0.78383 (13)	0.61519 (13)	0.0365 (6)	
H23A	0.2190	0.7838	0.6408	0.044*	
C24	0.2899 (2)	0.54610 (11)	0.40450 (12)	0.0270 (5)	
C25	0.3339 (2)	0.53717 (12)	0.33028 (12)	0.0321 (5)	
H25A	0.3739	0.5776	0.2986	0.039*	
C26	0.3207 (2)	0.47064 (14)	0.30157 (14)	0.0392 (6)	
H26A	0.3528	0.4654	0.2507	0.047*	
C27	0.2612 (3)	0.41201 (14)	0.34640 (16)	0.0491 (7)	
H27A	0.2505	0.3664	0.3268	0.059*	
C28	0.2175 (3)	0.41999 (15)	0.41987 (16)	0.0591 (9)	
H28A	0.1767	0.3795	0.4512	0.071*	
C29	0.2323 (3)	0.48634 (13)	0.44900 (14)	0.0449 (7)	
H29A	0.2024	0.4907	0.5001	0.054*	
C30	0.3020 (2)	0.61374 (11)	0.53389 (11)	0.0278 (5)	
C31	0.1744 (2)	0.60183 (12)	0.57780 (12)	0.0349 (5)	
H31A	0.1021	0.6058	0.5567	0.042*	
C32	0.1527 (3)	0.58431 (14)	0.65154 (13)	0.0449 (7)	
H32A	0.0659	0.5755	0.6807	0.054*	
C33	0.2570 (3)	0.57953 (14)	0.68288 (14)	0.0497 (7)	
H33A	0.2420	0.5675	0.7336	0.060*	
C34	0.3821 (3)	0.59211 (14)	0.64083 (14)	0.0440 (7)	
H34A	0.4536	0.5894	0.6627	0.053*	
C35	0.4051 (3)	0.60883 (12)	0.56653 (12)	0.0325 (5)	
H35A	0.4924	0.6170	0.5378	0.039*	
C36	0.4897 (2)	0.64966 (12)	0.39279 (11)	0.0264 (5)	
C37	0.5294 (2)	0.71156 (12)	0.34590 (12)	0.0305 (5)	
H37A	0.4660	0.7463	0.3382	0.037*	
C38	0.6605 (3)	0.72305 (14)	0.31034 (13)	0.0403 (6)	
H38A	0.6863	0.7653	0.2781	0.048*	
C39	0.7538 (2)	0.67351 (16)	0.32137 (14)	0.0434 (6)	
H39A	0.8441	0.6825	0.2985	0.052*	
C40	0.7150 (2)	0.61094 (14)	0.36573 (14)	0.0406 (6)	
H40A	0.7786	0.5759	0.3724	0.049*	
C41	0.5841 (2)	0.59860 (12)	0.40070 (12)	0.0320 (5)	
H41A	0.5585	0.5547	0.4305	0.038*	
C42	−0.0959 (2)	0.27369 (15)	0.06581 (15)	0.0430 (6)	
H42A	−0.1166	0.2703	0.0204	0.052*	
C43	−0.0740 (2)	0.21420 (14)	0.11211 (14)	0.0397 (6)	
H43A	−0.0727	0.1638	0.1019	0.048*	
C44	−0.0543 (2)	0.24217 (16)	0.17623 (14)	0.0448 (7)	
H44A	−0.0428	0.2142	0.2179	0.054*	
C45	−0.0551 (2)	0.31839 (17)	0.16664 (16)	0.0513 (8)	
H45A	−0.0401	0.3518	0.2001	0.062*	
C46	−0.0819 (2)	0.33820 (15)	0.09869 (17)	0.0489 (7)	
H46A	−0.0891	0.3868	0.0792	0.059*	
C47	0.3722 (2)	0.35360 (11)	0.10504 (11)	0.0253 (5)	
C48	0.3717 (2)	0.29229 (12)	0.15218 (12)	0.0312 (5)	
H48A	0.3012	0.2578	0.1614	0.037*	
C49	0.4720 (3)	0.28084 (15)	0.18576 (13)	0.0398 (6)	
H49A	0.4703	0.2386	0.2178	0.048*	
C50	0.5749 (3)	0.33051 (15)	0.17304 (13)	0.0415 (6)	
H50A	0.6452	0.3219	0.1951	0.050*	
C51	0.5748 (2)	0.39265 (14)	0.12811 (13)	0.0389 (6)	
H51A	0.6446	0.4275	0.1199	0.047*	
C52	0.4744 (2)	0.40456 (13)	0.09508 (12)	0.0320 (5)	
H52A	0.4746	0.4481	0.0650	0.038*	
C53	0.1809 (2)	0.45640 (11)	0.09877 (12)	0.0268 (5)	
C54	0.1394 (3)	0.46218 (13)	0.17349 (13)	0.0365 (6)	
H54A	0.1356	0.4193	0.2049	0.044*	
C55	0.1035 (3)	0.52857 (14)	0.20311 (14)	0.0427 (6)	
H55A	0.0742	0.5309	0.2545	0.051*	
C56	0.1098 (3)	0.59119 (15)	0.15896 (16)	0.0491 (7)	
H56A	0.0845	0.6370	0.1793	0.059*	
C57	0.1529 (4)	0.58706 (15)	0.08513 (17)	0.0614 (9)	
H57A	0.1591	0.6305	0.0542	0.074*	
C58	0.1876 (3)	0.52035 (13)	0.05509 (14)	0.0455 (7)	
H58A	0.2164	0.5184	0.0037	0.055*	
C59	0.3037 (2)	0.39087 (11)	−0.03316 (11)	0.0267 (5)	
C60	0.4359 (2)	0.39383 (12)	−0.06923 (12)	0.0303 (5)	
H60A	0.5001	0.3846	−0.0429	0.036*	
C61	0.4744 (3)	0.41027 (14)	−0.14399 (13)	0.0390 (6)	
H61A	0.5651	0.4119	−0.1685	0.047*	
C62	0.3830 (3)	0.42410 (14)	−0.18281 (13)	0.0425 (6)	
H62A	0.4105	0.4350	−0.2339	0.051*	
C63	0.2512 (3)	0.42223 (14)	−0.14764 (13)	0.0394 (6)	
H63A	0.1878	0.4326	−0.1743	0.047*	
C64	0.2118 (2)	0.40522 (13)	−0.07370 (12)	0.0332 (5)	
H64A	0.1209	0.4032	−0.0498	0.040*	
C65	0.2344 (2)	0.11055 (12)	0.06632 (12)	0.0284 (5)	
C66	0.1887 (2)	0.10033 (13)	0.14104 (13)	0.0348 (5)	
H66A	0.1373	0.1373	0.1677	0.042*	
C67	0.2169 (3)	0.03647 (14)	0.17766 (14)	0.0445 (6)	
H67A	0.1841	0.0300	0.2290	0.053*	
C68	0.2922 (3)	−0.01746 (14)	0.13984 (15)	0.0434 (6)	
H68A	0.3124	−0.0607	0.1651	0.052*	
C69	0.3378 (3)	−0.00837 (13)	0.06546 (15)	0.0417 (6)	
H69A	0.3889	−0.0457	0.0391	0.050*	
C70	0.3095 (2)	0.05498 (13)	0.02867 (13)	0.0350 (5)	
H70A	0.3414	0.0607	−0.0228	0.042*	
C71	0.1177 (2)	0.16815 (12)	−0.04709 (12)	0.0277 (5)	
C72	0.0650 (2)	0.09803 (13)	−0.04531 (13)	0.0363 (6)	
H72A	0.0784	0.0614	−0.0104	0.044*	
C73	−0.0067 (3)	0.08103 (14)	−0.09367 (15)	0.0458 (7)	
H73A	−0.0436	0.0332	−0.0910	0.055*	
C74	−0.0252 (3)	0.13294 (14)	−0.14578 (14)	0.0428 (6)	
H74A	−0.0731	0.1208	−0.1795	0.051*	
C75	0.0264 (2)	0.20256 (14)	−0.14848 (14)	0.0386 (6)	
H75A	0.0147	0.2385	−0.1845	0.046*	
C76	0.0952 (2)	0.22048 (13)	−0.09915 (13)	0.0344 (5)	
H76A	0.1279	0.2692	−0.1006	0.041*	
C77	0.3744 (2)	0.21120 (11)	−0.03924 (12)	0.0277 (5)	
C78	0.4077 (2)	0.21893 (14)	−0.11444 (13)	0.0378 (6)	
H78A	0.3405	0.2183	−0.1394	0.045*	
C79	0.5379 (3)	0.22765 (15)	−0.15421 (15)	0.0463 (7)	
H79A	0.5588	0.2330	−0.2059	0.056*	
C80	0.6362 (3)	0.22851 (15)	−0.11898 (15)	0.0457 (7)	
H80A	0.7250	0.2350	−0.1461	0.055*	
C81	0.6053 (2)	0.21992 (14)	−0.04404 (15)	0.0413 (6)	
H81A	0.6730	0.2199	−0.0194	0.050*	
C82	0.4760 (2)	0.21139 (13)	−0.00478 (13)	0.0333 (5)	
H82A	0.4559	0.2055	0.0468	0.040*	
Co1	0.18245 (3)	0.720773 (16)	0.417849 (15)	0.02465 (8)	
Co2	0.09119 (3)	0.279710 (16)	0.082317 (15)	0.02457 (8)	
P1	0.22712 (6)	0.80313 (3)	0.48282 (3)	0.02488 (12)	
P2	0.31370 (5)	0.63772 (3)	0.43712 (3)	0.02326 (12)	
P3	0.23336 (5)	0.36534 (3)	0.06398 (3)	0.02289 (12)	
P4	0.20477 (6)	0.19806 (3)	0.01871 (3)	0.02500 (12)	

### Atomic displacement parameters (Å^2^)

**Table:**

	*U*^11^	*U*^22^	*U*^33^	*U*^12^	*U*^13^	*U*^23^
C1	0.0464 (16)	0.0527 (16)	0.0326 (13)	0.0058 (12)	−0.0237 (11)	−0.0029 (11)
C2	0.0383 (14)	0.0403 (14)	0.0485 (15)	0.0106 (11)	−0.0261 (12)	−0.0060 (11)
C3	0.0248 (13)	0.0555 (16)	0.0491 (15)	0.0012 (12)	−0.0138 (11)	−0.0049 (13)
C4	0.0434 (16)	0.0385 (14)	0.0679 (18)	−0.0083 (12)	−0.0375 (14)	−0.0029 (13)
C5	0.0466 (16)	0.0560 (17)	0.0471 (15)	0.0160 (13)	−0.0309 (13)	−0.0255 (13)
C6	0.0295 (12)	0.0304 (12)	0.0275 (11)	0.0017 (9)	−0.0105 (9)	−0.0058 (9)
C7	0.0357 (14)	0.0382 (14)	0.0432 (14)	0.0077 (11)	−0.0059 (11)	0.0024 (11)
C8	0.0420 (16)	0.0404 (15)	0.0625 (18)	0.0156 (12)	−0.0038 (13)	−0.0042 (13)
C9	0.0329 (14)	0.0482 (16)	0.0462 (15)	0.0048 (12)	−0.0017 (11)	−0.0132 (12)
C10	0.0391 (15)	0.0448 (15)	0.0367 (13)	−0.0058 (12)	−0.0023 (11)	−0.0027 (11)
C11	0.0388 (14)	0.0290 (12)	0.0398 (13)	0.0031 (10)	−0.0044 (11)	−0.0056 (10)
C12	0.0258 (12)	0.0262 (11)	0.0348 (12)	0.0038 (9)	−0.0096 (9)	−0.0008 (9)
C13	0.0411 (15)	0.0298 (12)	0.0434 (14)	0.0001 (11)	−0.0179 (11)	−0.0028 (10)
C14	0.0432 (16)	0.0294 (13)	0.0687 (19)	−0.0019 (11)	−0.0241 (14)	−0.0014 (12)
C15	0.0348 (15)	0.0355 (14)	0.0679 (19)	−0.0033 (11)	−0.0104 (13)	0.0146 (13)
C16	0.0374 (15)	0.0481 (16)	0.0438 (15)	0.0014 (12)	−0.0065 (12)	0.0125 (12)
C17	0.0332 (13)	0.0353 (13)	0.0361 (13)	0.0035 (10)	−0.0099 (10)	0.0006 (10)
C18	0.0352 (13)	0.0189 (10)	0.0335 (12)	0.0012 (9)	−0.0148 (10)	−0.0037 (9)
C19	0.0373 (14)	0.0276 (12)	0.0402 (13)	0.0002 (10)	−0.0158 (11)	−0.0049 (10)
C20	0.0360 (15)	0.0355 (14)	0.0668 (18)	0.0025 (11)	−0.0248 (13)	−0.0052 (12)
C21	0.060 (2)	0.0394 (15)	0.0610 (18)	0.0009 (13)	−0.0427 (16)	−0.0012 (13)
C22	0.064 (2)	0.0446 (15)	0.0440 (15)	0.0033 (14)	−0.0314 (14)	−0.0003 (12)
C23	0.0443 (15)	0.0334 (13)	0.0356 (13)	0.0037 (11)	−0.0170 (11)	−0.0022 (10)
C24	0.0257 (12)	0.0250 (11)	0.0313 (11)	−0.0006 (9)	−0.0084 (9)	−0.0049 (9)
C25	0.0326 (13)	0.0287 (12)	0.0328 (12)	−0.0004 (10)	−0.0036 (10)	−0.0048 (9)
C26	0.0391 (15)	0.0421 (14)	0.0374 (13)	0.0033 (11)	−0.0091 (11)	−0.0139 (11)
C27	0.0609 (19)	0.0304 (13)	0.0584 (17)	−0.0041 (12)	−0.0169 (14)	−0.0154 (12)
C28	0.084 (2)	0.0305 (14)	0.0559 (18)	−0.0206 (14)	−0.0063 (16)	0.0004 (13)
C29	0.0617 (18)	0.0344 (14)	0.0339 (13)	−0.0109 (12)	−0.0036 (12)	−0.0011 (11)
C30	0.0364 (13)	0.0205 (10)	0.0259 (11)	0.0030 (9)	−0.0069 (9)	−0.0023 (8)
C31	0.0401 (14)	0.0306 (12)	0.0310 (12)	0.0050 (10)	−0.0039 (10)	−0.0023 (10)
C32	0.0588 (18)	0.0353 (14)	0.0303 (13)	0.0063 (12)	0.0064 (12)	0.0008 (10)
C33	0.080 (2)	0.0401 (15)	0.0275 (13)	0.0166 (14)	−0.0123 (14)	0.0006 (11)
C34	0.0662 (19)	0.0352 (14)	0.0361 (14)	0.0138 (13)	−0.0232 (13)	−0.0046 (11)
C35	0.0438 (15)	0.0238 (11)	0.0324 (12)	0.0058 (10)	−0.0140 (10)	−0.0040 (9)
C36	0.0264 (12)	0.0273 (11)	0.0264 (11)	−0.0014 (9)	−0.0073 (9)	−0.0061 (9)
C37	0.0325 (13)	0.0310 (12)	0.0280 (11)	−0.0009 (10)	−0.0082 (10)	0.0002 (9)
C38	0.0386 (15)	0.0456 (15)	0.0340 (13)	−0.0112 (12)	−0.0061 (11)	0.0052 (11)
C39	0.0254 (13)	0.0633 (18)	0.0392 (14)	−0.0071 (12)	−0.0036 (11)	−0.0057 (13)
C40	0.0296 (14)	0.0464 (15)	0.0475 (15)	0.0099 (11)	−0.0118 (11)	−0.0079 (12)
C41	0.0305 (13)	0.0285 (12)	0.0362 (12)	0.0020 (10)	−0.0072 (10)	−0.0021 (10)
C42	0.0196 (12)	0.0615 (17)	0.0478 (15)	−0.0042 (12)	−0.0093 (11)	−0.0006 (13)
C43	0.0256 (13)	0.0412 (14)	0.0474 (15)	−0.0113 (11)	−0.0001 (11)	−0.0032 (11)
C44	0.0299 (14)	0.0626 (18)	0.0346 (13)	−0.0076 (12)	0.0037 (11)	0.0024 (12)
C45	0.0238 (14)	0.0660 (19)	0.0571 (17)	−0.0069 (13)	0.0091 (12)	−0.0330 (15)
C46	0.0219 (13)	0.0410 (15)	0.075 (2)	0.0069 (11)	0.0032 (13)	−0.0006 (14)
C47	0.0242 (11)	0.0277 (11)	0.0243 (10)	0.0024 (9)	−0.0053 (9)	−0.0066 (9)
C48	0.0319 (13)	0.0340 (12)	0.0275 (11)	−0.0047 (10)	−0.0079 (10)	0.0004 (9)
C49	0.0434 (15)	0.0470 (15)	0.0322 (13)	0.0009 (12)	−0.0171 (11)	0.0045 (11)
C50	0.0349 (14)	0.0578 (17)	0.0369 (13)	0.0040 (12)	−0.0183 (11)	−0.0044 (12)
C51	0.0289 (13)	0.0473 (15)	0.0417 (14)	−0.0077 (11)	−0.0103 (11)	−0.0068 (11)
C52	0.0300 (13)	0.0311 (12)	0.0356 (12)	−0.0040 (10)	−0.0099 (10)	−0.0010 (10)
C53	0.0250 (12)	0.0256 (11)	0.0308 (11)	−0.0001 (9)	−0.0092 (9)	−0.0016 (9)
C54	0.0458 (15)	0.0290 (12)	0.0336 (13)	−0.0045 (11)	−0.0075 (11)	−0.0035 (10)
C55	0.0462 (16)	0.0410 (14)	0.0394 (14)	−0.0019 (12)	−0.0056 (12)	−0.0151 (11)
C56	0.0575 (18)	0.0347 (14)	0.0636 (18)	0.0151 (13)	−0.0273 (14)	−0.0191 (13)
C57	0.107 (3)	0.0284 (14)	0.0565 (18)	0.0152 (15)	−0.0359 (18)	−0.0006 (12)
C58	0.071 (2)	0.0315 (13)	0.0357 (14)	0.0070 (13)	−0.0168 (13)	0.0007 (11)
C59	0.0301 (12)	0.0248 (11)	0.0250 (11)	−0.0030 (9)	−0.0065 (9)	−0.0027 (8)
C60	0.0310 (13)	0.0286 (12)	0.0297 (12)	−0.0015 (10)	−0.0049 (10)	−0.0018 (9)
C61	0.0370 (14)	0.0426 (14)	0.0309 (12)	−0.0016 (11)	0.0019 (11)	0.0018 (10)
C62	0.0529 (17)	0.0464 (15)	0.0243 (12)	−0.0066 (12)	−0.0039 (11)	0.0024 (10)
C63	0.0465 (16)	0.0433 (14)	0.0316 (13)	−0.0086 (12)	−0.0171 (11)	0.0033 (10)
C64	0.0326 (13)	0.0360 (13)	0.0314 (12)	−0.0078 (10)	−0.0095 (10)	0.0010 (10)
C65	0.0248 (12)	0.0265 (11)	0.0362 (12)	−0.0033 (9)	−0.0123 (9)	−0.0007 (9)
C66	0.0391 (14)	0.0317 (12)	0.0358 (13)	−0.0020 (10)	−0.0133 (11)	−0.0026 (10)
C67	0.0578 (18)	0.0409 (15)	0.0402 (14)	−0.0072 (13)	−0.0235 (13)	0.0042 (11)
C68	0.0481 (16)	0.0302 (13)	0.0611 (17)	−0.0048 (12)	−0.0324 (14)	0.0060 (12)
C69	0.0331 (14)	0.0293 (13)	0.0652 (18)	0.0029 (10)	−0.0165 (12)	−0.0053 (12)
C70	0.0305 (13)	0.0348 (13)	0.0401 (13)	−0.0005 (10)	−0.0093 (10)	−0.0032 (10)
C71	0.0237 (12)	0.0290 (12)	0.0308 (11)	0.0000 (9)	−0.0071 (9)	−0.0043 (9)
C72	0.0391 (14)	0.0351 (13)	0.0380 (13)	−0.0092 (11)	−0.0176 (11)	0.0050 (10)
C73	0.0520 (17)	0.0374 (14)	0.0551 (16)	−0.0168 (12)	−0.0279 (13)	0.0034 (12)
C74	0.0446 (16)	0.0446 (15)	0.0471 (15)	−0.0064 (12)	−0.0263 (12)	−0.0017 (12)
C75	0.0396 (15)	0.0400 (14)	0.0398 (13)	0.0022 (11)	−0.0180 (11)	0.0024 (11)
C76	0.0366 (14)	0.0284 (12)	0.0406 (13)	−0.0023 (10)	−0.0144 (11)	−0.0018 (10)
C77	0.0261 (12)	0.0230 (11)	0.0329 (12)	0.0005 (9)	−0.0050 (9)	−0.0040 (9)
C78	0.0319 (14)	0.0466 (15)	0.0340 (13)	−0.0003 (11)	−0.0064 (10)	−0.0038 (11)
C79	0.0403 (16)	0.0561 (17)	0.0369 (14)	0.0023 (13)	0.0000 (12)	−0.0023 (12)
C80	0.0288 (14)	0.0478 (16)	0.0521 (16)	0.0012 (12)	0.0049 (12)	−0.0035 (12)
C81	0.0261 (13)	0.0438 (15)	0.0551 (16)	0.0017 (11)	−0.0112 (11)	−0.0073 (12)
C82	0.0290 (13)	0.0349 (13)	0.0351 (13)	0.0008 (10)	−0.0058 (10)	−0.0056 (10)
Co1	0.02439 (16)	0.02577 (15)	0.02583 (15)	0.00209 (12)	−0.00964 (12)	−0.00409 (12)
Co2	0.01960 (16)	0.02612 (15)	0.02680 (15)	−0.00197 (12)	−0.00350 (12)	−0.00282 (12)
P1	0.0264 (3)	0.0239 (3)	0.0257 (3)	0.0022 (2)	−0.0089 (2)	−0.0032 (2)
P2	0.0242 (3)	0.0225 (3)	0.0229 (3)	0.0000 (2)	−0.0056 (2)	−0.0020 (2)
P3	0.0224 (3)	0.0236 (3)	0.0225 (3)	−0.0010 (2)	−0.0056 (2)	−0.0013 (2)
P4	0.0230 (3)	0.0252 (3)	0.0271 (3)	−0.0013 (2)	−0.0066 (2)	−0.0026 (2)

### Geometric parameters (Å, °)

**Table:**

C83—C84	1.426 (5)	C34—C35	1.387 (3)
C83—H83A	0.9800	C34—H34A	0.9500
C83—H83B	0.9800	C35—H35A	0.9500
C83—H83C	0.9800	C36—C41	1.389 (3)
C85—C84	1.427 (5)	C36—C37	1.391 (3)
C85—C86	1.429 (5)	C36—P2	1.840 (2)
C85—H85A	0.9900	C37—C38	1.386 (3)
C85—H85B	0.9900	C37—H37A	0.9500
C86—C87	1.423 (12)	C38—C39	1.379 (4)
C86—H86A	0.9900	C38—H38A	0.9500
C86—H86B	0.9900	C39—C40	1.375 (4)
C87—C88	1.429 (5)	C39—H39A	0.9500
C87—H87A	0.9900	C40—C41	1.383 (3)
C87—H87B	0.9900	C40—H40A	0.9500
C88—H88A	0.9800	C41—H41A	0.9500
C88—H88B	0.9800	C42—C46	1.390 (4)
C88—H88C	0.9800	C42—C43	1.412 (4)
C84—C89	1.551 (8)	C42—Co2	2.082 (2)
C84—H84A	0.9900	C42—H42A	0.9500
C84—H84B	0.9900	C43—C44	1.414 (4)
C84—H84C	0.9800	C43—Co2	2.053 (2)
C84—H84D	0.9800	C43—H43A	0.9500
C84—H84E	0.9800	C44—C45	1.390 (4)
C89—C90	1.3900	C44—Co2	2.115 (2)
C89—C94	1.3900	C44—H44A	0.9500
C90—C91	1.3900	C45—C46	1.419 (4)
C90—H90	0.9500	C45—Co2	2.075 (2)
C91—C92	1.3900	C45—H45A	0.9500
C91—H91	0.9500	C46—Co2	2.072 (3)
C92—C93	1.3900	C46—H46A	0.9500
C92—H92	0.9500	C47—C48	1.389 (3)
C93—C94	1.3900	C47—C52	1.394 (3)
C93—H93	0.9500	C47—P3	1.835 (2)
C94—H94	0.9500	C48—C49	1.380 (3)
C1—C5	1.399 (4)	C48—H48A	0.9500
C1—C2	1.413 (4)	C49—C50	1.380 (4)
C1—Co1	2.114 (2)	C49—H49A	0.9500
C1—H1A	0.9500	C50—C51	1.377 (4)
C2—C3	1.412 (4)	C50—H50A	0.9500
C2—Co1	2.057 (2)	C51—C52	1.374 (3)
C2—H2A	0.9500	C51—H51A	0.9500
C3—C4	1.400 (4)	C52—H52A	0.9500
C3—Co1	2.085 (2)	C53—C58	1.381 (3)
C3—H3A	0.9500	C53—C54	1.387 (3)
C4—C5	1.419 (4)	C53—P3	1.853 (2)
C4—Co1	2.078 (2)	C54—C55	1.375 (3)
C4—H4A	0.9500	C54—H54A	0.9500
C5—Co1	2.077 (2)	C55—C56	1.367 (4)
C5—H5A	0.9500	C55—H55A	0.9500
C6—C7	1.390 (3)	C56—C57	1.368 (4)
C6—C11	1.396 (3)	C56—H56A	0.9500
C6—P1	1.843 (2)	C57—C58	1.382 (4)
C7—C8	1.391 (4)	C57—H57A	0.9500
C7—H7A	0.9500	C58—H58A	0.9500
C8—C9	1.374 (4)	C59—C60	1.387 (3)
C8—H8A	0.9500	C59—C64	1.401 (3)
C9—C10	1.377 (4)	C59—P3	1.839 (2)
C9—H9A	0.9500	C60—C61	1.390 (3)
C10—C11	1.380 (3)	C60—H60A	0.9500
C10—H10B	0.9500	C61—C62	1.371 (4)
C11—H11A	0.9500	C61—H61A	0.9500
C12—C17	1.389 (3)	C62—C63	1.379 (4)
C12—C13	1.394 (3)	C62—H62A	0.9500
C12—P1	1.847 (2)	C63—C64	1.378 (3)
C13—C14	1.386 (3)	C63—H63A	0.9500
C13—H13A	0.9500	C64—H64A	0.9500
C14—C15	1.373 (4)	C65—C66	1.381 (3)
C14—H14A	0.9500	C65—C70	1.397 (3)
C15—C16	1.368 (4)	C65—P4	1.851 (2)
C15—H15A	0.9500	C66—C67	1.391 (3)
C16—C17	1.387 (3)	C66—H66A	0.9500
C16—H16A	0.9500	C67—C68	1.377 (4)
C17—H17A	0.9500	C67—H67A	0.9500
C18—C23	1.387 (3)	C68—C69	1.373 (4)
C18—C19	1.392 (3)	C68—H68A	0.9500
C18—P1	1.845 (2)	C69—C70	1.385 (3)
C19—C20	1.382 (3)	C69—H69A	0.9500
C19—H19A	0.9500	C70—H70A	0.9500
C20—C21	1.381 (4)	C71—C72	1.391 (3)
C20—H20A	0.9500	C71—C76	1.397 (3)
C21—C22	1.366 (4)	C71—P4	1.841 (2)
C21—H21A	0.9500	C72—C73	1.382 (3)
C22—C23	1.383 (4)	C72—H72A	0.9500
C22—H22A	0.9500	C73—C74	1.379 (4)
C23—H23A	0.9500	C73—H73A	0.9500
C24—C29	1.380 (3)	C74—C75	1.376 (3)
C24—C25	1.389 (3)	C74—H74A	0.9500
C24—P2	1.856 (2)	C75—C76	1.380 (3)
C25—C26	1.382 (3)	C75—H75A	0.9500
C25—H25A	0.9500	C76—H76A	0.9500
C26—C27	1.373 (4)	C77—C78	1.382 (3)
C26—H26A	0.9500	C77—C82	1.393 (3)
C27—C28	1.372 (4)	C77—P4	1.854 (2)
C27—H27A	0.9500	C78—C79	1.392 (3)
C28—C29	1.386 (4)	C78—H78A	0.9500
C28—H28A	0.9500	C79—C80	1.373 (4)
C29—H29A	0.9500	C79—H79A	0.9500
C30—C35	1.385 (3)	C80—C81	1.379 (4)
C30—C31	1.400 (3)	C80—H80A	0.9500
C30—P2	1.840 (2)	C81—C82	1.381 (3)
C31—C32	1.381 (3)	C81—H81A	0.9500
C31—H31A	0.9500	C82—H82A	0.9500
C32—C33	1.380 (4)	Co1—P2	2.1246 (6)
C32—H32A	0.9500	Co1—P1	2.1296 (6)
C33—C34	1.368 (4)	Co2—P3	2.1204 (6)
C33—H33A	0.9500	Co2—P4	2.1371 (6)
			
C84—C83—H83A	109.5	C41—C40—H40A	119.8
C84—C83—H83B	109.5	C40—C41—C36	120.8 (2)
H83A—C83—H83B	109.5	C40—C41—H41A	119.6
C84—C83—H83C	109.5	C36—C41—H41A	119.6
H83A—C83—H83C	109.5	C46—C42—C43	107.7 (2)
H83B—C83—H83C	109.5	C46—C42—Co2	70.09 (15)
C84—C85—C86	117.5 (7)	C43—C42—Co2	68.96 (14)
C84—C85—H85A	107.9	C46—C42—H42A	126.2
C86—C85—H85A	107.9	C43—C42—H42A	126.2
C84—C85—H85B	107.9	Co2—C42—H42A	126.4
C86—C85—H85B	107.9	C42—C43—C44	108.6 (2)
H85A—C85—H85B	107.2	C42—C43—Co2	71.13 (14)
C87—C86—C85	126.4 (8)	C44—C43—Co2	72.54 (14)
C87—C86—H86A	105.7	C42—C43—H43A	125.7
C85—C86—H86A	105.7	C44—C43—H43A	125.7
C87—C86—H86B	105.7	Co2—C43—H43A	122.3
C85—C86—H86B	105.7	C45—C44—C43	106.8 (2)
H86A—C86—H86B	106.2	C45—C44—Co2	69.08 (14)
C86—C87—C88	117.3 (9)	C43—C44—Co2	67.84 (13)
C86—C87—H87A	108.0	C45—C44—H44A	126.6
C88—C87—H87A	108.0	C43—C44—H44A	126.6
C86—C87—H87B	108.0	Co2—C44—H44A	128.0
C88—C87—H87B	108.0	C44—C45—C46	109.0 (2)
H87A—C87—H87B	107.2	C44—C45—Co2	72.18 (14)
C87—C88—H88A	109.5	C46—C45—Co2	69.89 (14)
C87—C88—H88B	109.5	C44—C45—H45A	125.5
H88A—C88—H88B	109.5	C46—C45—H45A	125.5
C87—C88—H88C	109.5	Co2—C45—H45A	124.0
H88A—C88—H88C	109.5	C42—C46—C45	107.7 (2)
H88B—C88—H88C	109.5	C42—C46—Co2	70.81 (15)
C83—C84—C85	118.6 (6)	C45—C46—Co2	70.09 (15)
C83—C84—C89	145.0 (7)	C42—C46—H46A	126.1
C83—C84—H84A	107.7	C45—C46—H46A	126.1
C85—C84—H84A	107.7	Co2—C46—H46A	124.6
C89—C84—H84A	96.8	C48—C47—C52	118.0 (2)
C83—C84—H84B	107.7	C48—C47—P3	117.96 (17)
C85—C84—H84B	107.7	C52—C47—P3	123.96 (17)
C89—C84—H84B	87.8	C49—C48—C47	120.9 (2)
H84A—C84—H84B	107.1	C49—C48—H48A	119.6
C83—C84—H84C	104.1	C47—C48—H48A	119.6
C85—C84—H84C	136.8	C48—C49—C50	120.3 (2)
C89—C84—H84C	109.5	C48—C49—H49A	119.9
H84A—C84—H84C	61.6	C50—C49—H49A	119.9
H84B—C84—H84C	49.0	C51—C50—C49	119.5 (2)
C83—C84—H84D	66.7	C51—C50—H50A	120.2
C85—C84—H84D	93.8	C49—C50—H50A	120.2
C89—C84—H84D	109.5	C52—C51—C50	120.4 (2)
H84A—C84—H84D	57.4	C52—C51—H51A	119.8
H84B—C84—H84D	157.2	C50—C51—H51A	119.8
H84C—C84—H84D	109.5	C51—C52—C47	120.9 (2)
C83—C84—H84E	48.1	C51—C52—H52A	119.5
C85—C84—H84E	95.4	C47—C52—H52A	119.5
C89—C84—H84E	109.5	C58—C53—C54	117.3 (2)
H84A—C84—H84E	153.6	C58—C53—P3	124.11 (18)
H84B—C84—H84E	76.9	C54—C53—P3	118.46 (16)
H84C—C84—H84E	109.5	C55—C54—C53	121.5 (2)
H84D—C84—H84E	109.5	C55—C54—H54A	119.2
C90—C89—C94	120.0	C53—C54—H54A	119.2
C90—C89—C84	115.8 (6)	C56—C55—C54	120.3 (2)
C94—C89—C84	124.2 (6)	C56—C55—H55A	119.9
C91—C90—C89	120.0	C54—C55—H55A	119.9
C91—C90—H90	120.0	C55—C56—C57	119.2 (2)
C89—C90—H90	120.0	C55—C56—H56A	120.4
C90—C91—C92	120.0	C57—C56—H56A	120.4
C90—C91—H91	120.0	C56—C57—C58	120.7 (3)
C92—C91—H91	120.0	C56—C57—H57A	119.7
C91—C92—C93	120.0	C58—C57—H57A	119.7
C91—C92—H92	120.0	C53—C58—C57	120.9 (2)
C93—C92—H92	120.0	C53—C58—H58A	119.5
C94—C93—C92	120.0	C57—C58—H58A	119.5
C94—C93—H93	120.0	C60—C59—C64	118.4 (2)
C92—C93—H93	120.0	C60—C59—P3	126.43 (18)
C93—C94—C89	120.0	C64—C59—P3	115.06 (17)
C93—C94—H94	120.0	C59—C60—C61	120.0 (2)
C89—C94—H94	120.0	C59—C60—H60A	120.0
C5—C1—C2	106.7 (2)	C61—C60—H60A	120.0
C5—C1—Co1	69.05 (14)	C62—C61—C60	120.8 (2)
C2—C1—Co1	68.04 (13)	C62—C61—H61A	119.6
C5—C1—H1A	126.6	C60—C61—H61A	119.6
C2—C1—H1A	126.6	C61—C62—C63	120.0 (2)
Co1—C1—H1A	127.8	C61—C62—H62A	120.0
C3—C2—C1	109.1 (2)	C63—C62—H62A	120.0
C3—C2—Co1	71.14 (14)	C64—C63—C62	119.8 (2)
C1—C2—Co1	72.38 (14)	C64—C63—H63A	120.1
C3—C2—H2A	125.5	C62—C63—H63A	120.1
C1—C2—H2A	125.5	C63—C64—C59	121.0 (2)
Co1—C2—H2A	122.6	C63—C64—H64A	119.5
C4—C3—C2	107.4 (2)	C59—C64—H64A	119.5
C4—C3—Co1	70.07 (14)	C66—C65—C70	118.2 (2)
C2—C3—Co1	69.02 (14)	C66—C65—P4	120.40 (17)
C4—C3—H3A	126.3	C70—C65—P4	121.32 (17)
C2—C3—H3A	126.3	C65—C66—C67	120.8 (2)
Co1—C3—H3A	126.1	C65—C66—H66A	119.6
C3—C4—C5	107.9 (2)	C67—C66—H66A	119.6
C3—C4—Co1	70.63 (14)	C68—C67—C66	120.4 (2)
C5—C4—Co1	69.97 (14)	C68—C67—H67A	119.8
C3—C4—H4A	126.1	C66—C67—H67A	119.8
C5—C4—H4A	126.1	C69—C68—C67	119.6 (2)
Co1—C4—H4A	124.9	C69—C68—H68A	120.2
C1—C5—C4	108.8 (2)	C67—C68—H68A	120.2
C1—C5—Co1	71.96 (14)	C68—C69—C70	120.3 (2)
C4—C5—Co1	70.08 (14)	C68—C69—H69A	119.9
C1—C5—H5A	125.6	C70—C69—H69A	119.9
C4—C5—H5A	125.6	C69—C70—C65	120.8 (2)
Co1—C5—H5A	124.0	C69—C70—H70A	119.6
C7—C6—C11	117.8 (2)	C65—C70—H70A	119.6
C7—C6—P1	124.03 (18)	C72—C71—C76	117.7 (2)
C11—C6—P1	118.06 (17)	C72—C71—P4	124.18 (17)
C6—C7—C8	120.5 (2)	C76—C71—P4	117.97 (17)
C6—C7—H7A	119.7	C73—C72—C71	120.9 (2)
C8—C7—H7A	119.7	C73—C72—H72A	119.6
C9—C8—C7	120.5 (2)	C71—C72—H72A	119.6
C9—C8—H8A	119.7	C74—C73—C72	120.6 (2)
C7—C8—H8A	119.7	C74—C73—H73A	119.7
C8—C9—C10	119.7 (2)	C72—C73—H73A	119.7
C8—C9—H9A	120.1	C75—C74—C73	119.4 (2)
C10—C9—H9A	120.1	C75—C74—H74A	120.3
C9—C10—C11	120.0 (2)	C73—C74—H74A	120.3
C9—C10—H10B	120.0	C74—C75—C76	120.3 (2)
C11—C10—H10B	120.0	C74—C75—H75A	119.8
C10—C11—C6	121.3 (2)	C76—C75—H75A	119.8
C10—C11—H11A	119.4	C75—C76—C71	121.1 (2)
C6—C11—H11A	119.4	C75—C76—H76A	119.5
C17—C12—C13	118.4 (2)	C71—C76—H76A	119.5
C17—C12—P1	120.35 (18)	C78—C77—C82	117.6 (2)
C13—C12—P1	121.01 (17)	C78—C77—P4	124.57 (19)
C14—C13—C12	120.7 (2)	C82—C77—P4	117.79 (17)
C14—C13—H13A	119.7	C77—C78—C79	121.1 (3)
C12—C13—H13A	119.7	C77—C78—H78A	119.4
C15—C14—C13	120.0 (3)	C79—C78—H78A	119.4
C15—C14—H14A	120.0	C80—C79—C78	120.2 (3)
C13—C14—H14A	120.0	C80—C79—H79A	119.9
C16—C15—C14	120.0 (2)	C78—C79—H79A	119.9
C16—C15—H15A	120.0	C79—C80—C81	119.6 (2)
C14—C15—H15A	120.0	C79—C80—H80A	120.2
C15—C16—C17	120.7 (2)	C81—C80—H80A	120.2
C15—C16—H16A	119.7	C80—C81—C82	120.0 (3)
C17—C16—H16A	119.7	C80—C81—H81A	120.0
C16—C17—C12	120.2 (2)	C82—C81—H81A	120.0
C16—C17—H17A	119.9	C81—C82—C77	121.4 (2)
C12—C17—H17A	119.9	C81—C82—H82A	119.3
C23—C18—C19	117.9 (2)	C77—C82—H82A	119.3
C23—C18—P1	124.55 (19)	C2—Co1—C5	66.18 (10)
C19—C18—P1	117.55 (17)	C2—Co1—C4	66.42 (10)
C20—C19—C18	121.2 (2)	C5—Co1—C4	39.95 (11)
C20—C19—H19A	119.4	C2—Co1—C3	39.83 (10)
C18—C19—H19A	119.4	C5—Co1—C3	66.41 (11)
C21—C20—C19	119.8 (3)	C4—Co1—C3	39.30 (10)
C21—C20—H20A	120.1	C2—Co1—C1	39.58 (10)
C19—C20—H20A	120.1	C5—Co1—C1	38.99 (10)
C22—C21—C20	119.5 (3)	C4—Co1—C1	66.27 (11)
C22—C21—H21A	120.2	C3—Co1—C1	66.44 (11)
C20—C21—H21A	120.2	C2—Co1—P2	166.40 (7)
C21—C22—C23	120.9 (3)	C5—Co1—P2	100.57 (8)
C21—C22—H22A	119.5	C4—Co1—P2	101.69 (8)
C23—C22—H22A	119.5	C3—Co1—P2	133.54 (8)
C22—C23—C18	120.6 (3)	C1—Co1—P2	130.84 (7)
C22—C23—H23A	119.7	C2—Co1—P1	94.02 (7)
C18—C23—H23A	119.7	C5—Co1—P1	156.20 (8)
C29—C24—C25	117.9 (2)	C4—Co1—P1	144.99 (9)
C29—C24—P2	124.46 (17)	C3—Co1—P1	107.39 (8)
C25—C24—P2	117.60 (16)	C1—Co1—P1	117.21 (8)
C26—C25—C24	121.3 (2)	P2—Co1—P1	99.57 (2)
C26—C25—H25A	119.4	C43—Co2—C46	66.49 (11)
C24—C25—H25A	119.4	C43—Co2—C45	66.10 (10)
C27—C26—C25	120.1 (2)	C46—Co2—C45	40.02 (12)
C27—C26—H26A	119.9	C43—Co2—C42	39.91 (10)
C25—C26—H26A	119.9	C46—Co2—C42	39.11 (11)
C28—C27—C26	119.3 (2)	C45—Co2—C42	66.18 (11)
C28—C27—H27A	120.4	C43—Co2—C44	39.63 (10)
C26—C27—H27A	120.4	C46—Co2—C44	66.19 (11)
C27—C28—C29	120.8 (2)	C45—Co2—C44	38.74 (11)
C27—C28—H28A	119.6	C42—Co2—C44	66.30 (11)
C29—C28—H28A	119.6	C43—Co2—P3	167.16 (8)
C24—C29—C28	120.6 (2)	C46—Co2—P3	101.83 (8)
C24—C29—H29A	119.7	C45—Co2—P3	101.65 (8)
C28—C29—H29A	119.7	C42—Co2—P3	133.01 (8)
C35—C30—C31	118.3 (2)	C44—Co2—P3	132.03 (8)
C35—C30—P2	126.51 (18)	C43—Co2—P4	93.12 (7)
C31—C30—P2	115.13 (18)	C46—Co2—P4	143.73 (9)
C32—C31—C30	120.6 (3)	C45—Co2—P4	155.80 (9)
C32—C31—H31A	119.7	C42—Co2—P4	106.35 (8)
C30—C31—H31A	119.7	C44—Co2—P4	117.11 (8)
C33—C32—C31	120.1 (3)	P3—Co2—P4	99.67 (2)
C33—C32—H32A	119.9	C6—P1—C18	102.02 (10)
C31—C32—H32A	119.9	C6—P1—C12	102.62 (10)
C34—C33—C32	120.0 (2)	C18—P1—C12	96.25 (10)
C34—C33—H33A	120.0	C6—P1—Co1	110.41 (7)
C32—C33—H33A	120.0	C18—P1—Co1	124.20 (7)
C33—C34—C35	120.4 (3)	C12—P1—Co1	118.20 (7)
C33—C34—H34A	119.8	C36—P2—C30	105.96 (10)
C35—C34—H34A	119.8	C36—P2—C24	98.20 (10)
C30—C35—C34	120.6 (2)	C30—P2—C24	100.97 (10)
C30—C35—H35A	119.7	C36—P2—Co1	118.68 (7)
C34—C35—H35A	119.7	C30—P2—Co1	114.65 (7)
C41—C36—C37	118.3 (2)	C24—P2—Co1	115.79 (7)
C41—C36—P2	123.68 (17)	C47—P3—C59	106.47 (10)
C37—C36—P2	117.99 (17)	C47—P3—C53	97.20 (10)
C38—C37—C36	120.6 (2)	C59—P3—C53	100.74 (10)
C38—C37—H37A	119.7	C47—P3—Co2	118.37 (7)
C36—C37—H37A	119.7	C59—P3—Co2	113.12 (7)
C39—C38—C37	120.4 (2)	C53—P3—Co2	118.42 (7)
C39—C38—H38A	119.8	C71—P4—C65	103.07 (10)
C37—C38—H38A	119.8	C71—P4—C77	101.79 (10)
C40—C39—C38	119.4 (2)	C65—P4—C77	96.51 (10)
C40—C39—H39A	120.3	C71—P4—Co2	109.03 (7)
C38—C39—H39A	120.3	C65—P4—Co2	117.94 (7)
C39—C40—C41	120.5 (2)	C77—P4—Co2	125.42 (7)
C39—C40—H40A	119.8		
